# Avian Influenza Surveillance Among Migratory Birds, Poultry, and Humans Around Nansi Lake, China, 2021–2024

**DOI:** 10.3390/v17081117

**Published:** 2025-08-14

**Authors:** Sheng Zhang, Yu-Min Liang, Dong-Mei Wang, Chao Shang, Wang-Qian Wei, Xin-Jing Zhao, Li-Bo Li, Wen-Guo Jiang, Bao-Jin Guo, Bo-Yan Jiao, Jun Ma, Yun-Bo Qiu, Yong-Biao Cui, Guo-Qiang Wang, Jin-Jin Chen, Qiang Xu, Chen-Long Lv, Feng Hong, Guo-Lin Wang, Li-Qun Fang

**Affiliations:** 1 The Key Laboratory of Environmental Pollution Monitoring and Disease Control, School of Public Health, Ministry of Education, Guizhou Medical University, Guiyang 550025, China; 2 State Key Laboratory of Pathogen and Biosecurity, Academy of Military Medical Sciences, Beijing 100071, China; 3 Jining Center for Disease Control and Prevention, Jining 272000, China; 4 Dezhou Center for Disease Control and Prevention, Dezhou 253016, China; 5 Changchun Veterinary Research Institute, Chinese Academy of Agricultural Sciences, Changchun 130122, China; 6 Department of Epidemiology and Health Statistics, School of Public Health, Anhui Medical University, Hefei 230032, China; 7 Nansi Lake Nature Reserve, Jining 272100, China

**Keywords:** avian influenza A viruses, migratory birds, poultry, avian-exposed population, genomic reassortment, serological investigation

## Abstract

Avian influenza A viruses (AIVs) pose a significant pandemic threat due to their cross-species transmission potential. However, AIV surveillance at the critical “migratory birds–poultry-exposed population” interface remains limited. Between 2021 and 2024, we implemented a prospective One Health surveillance program around Nansi Lake, monitoring AIVs in migratory birds, poultry, and environmental samples, as well as serological investigations against representative AIVs among migratory birds or poultry-exposed subjects. AIVs were detected in 2.1% (30/1417) of migratory bird samples and 10.2% (100/978) of poultry samples. Among these, we identified ten highly pathogenic avian influenza (HPAI) H5 subtype viruses, one HPAI H7N9 virus, and five low pathogenic avian influenza (LPAI) H9N2 viruses. Phylogenetic analysis revealed evidence of frequent genomic reassortment events involving H5 subtype viruses among migratory birds, poultry, and humans. Serological investigation also suggested that both migratory birds and the poultry-exposed population had a higher risk of getting AIV infection than the general control population, especially against the H9N2 virus. Our study emphasizes the importance of strengthening continuous prospective surveillance of AIVs among migratory birds, poultry, and their exposed individuals to prevent and control potential outbreaks.

## 1. Introduction

Avian influenza A viruses (AIVs) have emerged as a critical global public health and economic challenge, with their prevalence demonstrating a concerning upward trajectory in recent decades [1,2]. Between January 2013 and June 2022, outbreaks involving 34 AIV subtypes resulted in over 300 million bird deaths or culling across 21,249 reported incidents [1]. As of June 2023, the WHO had documented human infections in China caused by nine subtypes. Notably, five of these subtypes—H3N8, H6N1, H7N4, H10N3, and H10N8—have been reported exclusively in China [3].

Migratory birds serve as essential reservoirs and vectors for the global dissemination and genetic reassortment of AIVs [4,5]. The evolutionary lineage of contemporary HPAI H5 subtypes can be traced to the prototype A/Goose/Guangdong/1/96 H5N1 strain first identified in China [6]. Since 2005, the HPAI H5N1 viruses have repeatedly spilled over into migratory birds, facilitating episodic spread across Asia, Europe, Africa, and, more recently, North and South America [6,7,8,9]. Furthermore, since 2014, HPAI H5N1 viruses bearing H5 hemagglutinin (HA) genes from genetic clade 2.3.4.4 have driven severe outbreaks across Africa, Europe, Asia, and North America [10]. The threat expanded significantly during the 2020–2023 panzootic, with natural H5N1 infections documented in over 48 mammal species [3]. Most alarmingly, in 2024, HPAI H5N1 viruses were detected in dairy cattle, domestic cats, and exposed farm workers [11,12]. Thus, HPAI H5 subtype viruses remain a devastating threat to the global poultry industry and pose a growing threat to human health.

Live poultry markets (LPMs) constitute a critical nexus for AIV reassortment and spillover into human populations [13,14,15]. The presence of diverse poultry species within retail LPMs amplifies the genetic diversity of circulating AIVs, potentially fostering the emergence of novel AIV subtypes posing significant public health threats [16]. Epidemiology data from the WHO indicate that approximately 75% of human AIV infections involve documented animal exposure, with LPM visits representing the predominant risk factor [1,17]. Over recent decades, novel AIV subtypes, including H6N1, H7N9, H5N6, H10N3, H10N8, and H3N8, have caused repeated human infections in China, with transmission events consistently associated with LPM exposure [18,19,20,21,22,23,24].

Serological evidence demonstrates significantly higher H9N2 seroprevalence (5.6–50.3%) among poultry-exposed populations compared with lower rates (0.8–3.9%) for H5 and H7 subtypes [25,26,27,28]. Furthermore, poultry workers have a higher risk of getting infections of AIVs than the general control population [25]. In contrast, serological data on migratory bird-exposed populations remain scarce. Only one study in Malaysia has reported an association between H5-specific binding antibody levels and migratory bird distribution [29]. Consequently, expanded serological investigations are essential to accurately assess AIV infection risks among human populations residing near migratory bird habitats.

Most prior studies of AIVs have focused separately on migratory birds, poultry, or humans, limiting our ability to fully understand cross-species transmission dynamics. Nansi Lake (116°34′09″~117°21′53″ E, 34°23′56″~35°17′39″ N), one of the largest freshwater lakes in northern China, lies along the globally significant “East Asia–Australasia” migratory flyway. Herein, we enrolled migratory birds, poultry, and humans within the Nansi Lake ecosystem to explore the AIV evolution, transmission patterns, and infection risks across this interconnected small-scale ecosystem.

## 2. Materials and Methods

### 2.1. Study Objects, Sampling, and Data Collection

**Figure 1:**
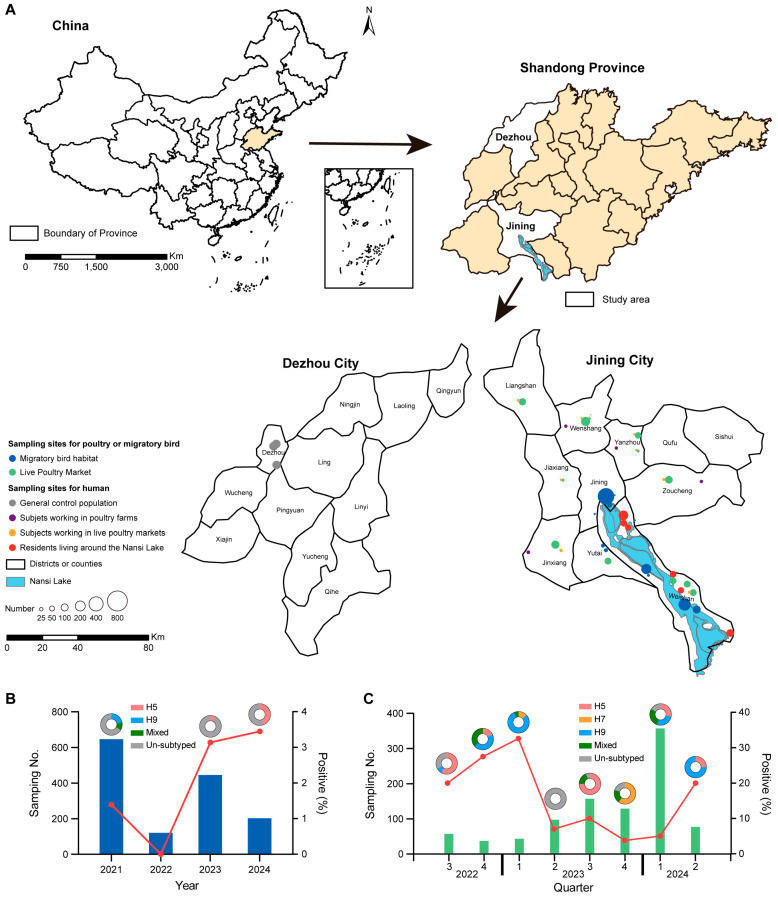
Sampling sites and prevalence of avian influenza virus among poultry and migratory birds. (**A**) Geographic distribution of sampling sites for poultry, migratory birds, and humans. (**B**) Prevalence of avian influenza virus in fecal samples from migratory birds. (**C**) Prevalence of avian influenza virus in poultry samples collected from live poultry markets.

Between June 2021 and June 2024, fecal samples were collected from the migratory birds at various sites around Nansi Lake, with sampling locations recorded by latitude and longitude (Figure 1A and Supplemental Table S1). Meanwhile, poultry samples (feces, water, cage swabs, and chopping board swabs) were collected from different LPMs surrounding Nansi Lake across Jining city counties between July 2022 and June 2024 (Figure 1A and Supplemental Table S1). All biological samples were immediately placed in sterile tubes containing 3 mL viral transport medium and transported to the laboratory under cold chain conditions. Additionally, 5 mL blood samples and demographic information were collected from four occupational groups: workers in poultry farms, workers in LPMs, residents living within 3 km of migratory birds at Nansi Lake in Jining city, and a general control population in Dezhou city with no history of avian exposure during the last 12 months preceding sample collection (Figure 1A and Supplemental Table S1).

### 2.2. Molecular Detection, Isolation, and Sequencing

Viral RNA was extracted from all collected samples using the Nucleic Acid Extraction Kit (TaKaRa, Shiga, Japan, Cat No. 9766). AIVs were screened via quantitative reverse transcription PCR (qRT-PCR) targeting the influenza matrix gene segment using the one-step RT-PCR kit (TaKaRa, Cat. No. 064A) on a LightCycler 480 Real-Time PCR System (Roche, Basel, Switzerland). Each run included a negative control and a viral RNA positive control. Samples with Ct values less than or equal to 38 were considered AIV-positive. Then AIV-positive samples were further subtyped using the Avian Influenza Virus H5/H7/H9 RNA Subtyping Kit (Daan Gene, Guangzhou, China, Cat. No. DS0180). Positive samples with Ct < 35 were selected for further isolation using 9- to 11-day-old specific pathogen-free (SPF) chicken embryos following the documented standard protocols. The presence of AIV in the allantoic fluid was confirmed by both RT-PCR and hemagglutination (HA) assays. The whole genome of positive samples was amplified by universal primers [30,31] with the one-step RT-PCR Kit (TAKARA, Shiga, JapanCat. No. 057A) and then sequenced using standard techniques on an Applied Biosystems 3730 xl DNA Analyzer (Foster City, CA, USA) using the Big Dye Terminator Kit v3.1 (Applied Biosystems). Consensus sequences were deposited into the GISAID EpiFlu^TM^ database (https://www.gisaid.org), with accession numbers provided in Supplemental Table S4.

### 2.3. Phylogenetic and Reassortment Analysis

Sequences were assembled by using SeqMan Lasergene version 7 (DNASTAR). Representative sequences of H5, H7, and H9 subtype viruses were downloaded from the database of GenBank and GISAID. Multiple sequence alignments and phylogenetic trees were constructed by MEGA version 10 (https://www.megasoftware.net/dload_win_gui, accessed on 1 June 2025) applying the maximum-likelihood method with 1000 bootstrap replicates. For clade classification, H9 subtype viruses were categorized into lineages (Y, B, and G) following previously established criteria [32]. Similarly, H5 subtype viruses were classified into clades 2.3.4.a–h based on reported studies [33,34]. To assess potential gene reassortment events, we determined the genomic origins of the identified viruses by analyzing individual gene phylogenies and reconstructing their recombination patterns.

### 2.4. Hemagglutination Inhibition (HI) Assay

All serum samples were initially screened using the hemagglutination inhibition (HI) assay [35]. Considering the high potential risk for spillover and the availability of the AIV subtypes, the following isolated strains were used for the HI and the following MN assays: clade 2.3.2.1c H5N1 (A/Chicken/Shandong/WS55/2014), clade 2.3.4.4b H5N2 (A/Chicken/Shandong/WS17/2022), clade 2.3.4.4b H5N6 (A/Egret/Shandong/JN529/2023), H7N9 (A/Chicken/Shandong/WS75/2022), and clade B4.6 H9N2 (A/Chicken/Shandong/WS04/2022). The serum for HI assay was pretreated with receptor-destroying enzyme (Denka Seiken Co., Ltd., Tokyo, Japan) at 37 °C for 18 h, followed by heat inactivation at 56 °C for 30 min to eliminate non-specific hemagglutination factors. Two-fold serial dilutions of serum (from 1:10 to 1:160) were incubated with 4 hemagglutinin (HA) units of each viral antigen using 1% horse red blood cells (for H5N1, H5N2, H5N6, and H7N9) or 1% chicken red blood cells (for H9N2) based on the different receptor specificity. The HI titers were defined as the reciprocal of the highest serum dilution that completely inhibited hemagglutination. A seropositive result was defined as an HI titer ≥ 1:40 for all tested strains.

### 2.5. Microneutralization (MN) Assay

Serum samples exhibiting an HI titer ≥ 1:10 were further identified by the microneutralization (MN) assay [36]. Briefly, 100 μL MDCK cell suspension with 3.0 × 10^4^ cells was added to each well of the plate and then incubated at 37 °C with 5% CO_2_ for 24 h. Subsequently, two-fold serial dilutions of serum ranging from 1:10 to 1:320 were incubated with 100 × 50% tissue culture infective dose (TCID_50_) of each tested virus at 37 °C for 2 h, and then the serum–virus mixtures were transferred to the cell plates and incubated under the same conditions for 24 h. Following incubation, the cells were washed twice with PBS and fixed with 100 μL of pre-cooled 80% acetone for 10 min at room temperature. After fixation and an additional PBS wash, 100 μL of diluted primary anti-influenza virus nucleoprotein monoclonal antibody (Merck, Darmstadt, Germany, Cat No. MAB8257 and MAB8258, 1:8000) was added to each well and incubated at 37 °C for 30 min. Plates were then washed and incubated with 100 μL of diluted HRP-labeled goat anti-mouse IgG antibody (ZSGB-BIO, Shanghai, China, Cat No. ZB2305, 1:1500) at 37 °C for 45 min. After a final washing step, 100 μL of TMB substrate (Mei5bio, Beijing, China, Cat No. MF142-01) was added, and the plate was incubated in the dark at 37 °C for 5 min. The reaction was stopped by adding 100 μL of stop solution (Solarbio, Beijing, China, Cat. No. C1058). The MN titer was expressed as the reciprocal of the highest dilution of serum with optical density (OD) < X, where X = [(average OD of virus control wells) − (average OD of cell control wells)]/2 + average OD of cell control wells. The MN titer is the reciprocal of the highest serum dilution that yielded > 50% neutralization. Samples with titers < 1:10, we assigned a value of 1:5 for analysis. A seropositive result was defined as an MN titer ≥ 1:40 for all tested strains.

### 2.6. Statistical Analysis

We performed all statistical analyses using GraphPad Prism 8.1.2 software (https://www.graphpad.com) and SPSS Statistics 23 (IBM, Armonk, New York, NY, USA, https://www.ibm.com). Statistical differences between different groups were tested using either Pearson’s χ^2^ test or Fisher’s exact test. A two-tailed *p*-value ≤ 0.05 was considered statistically significant.

### 2.7. Biosafety Statement and Ethical Considerations

All procedures related to virus isolation, diagnostics, and experiments involving HPAI H5 and H7N9 viruses were carried out in a Biosafety Level 3 (BSL-3) facility and were approved by the Changchun Veterinary Research Institute of the Chinese Academy of Agricultural Sciences. The enrollment of participants was approved by the Institutional Review Boards of the Academy of Military Medical Sciences (IRB numbers: AF/SC-08/02.100, and AF/SC-08/02.245) and informed consent was obtained from each of the participants.

## 3. Results

### 3.1. Prevalence and Distribution of AIVs Among Migratory Birds and Poultry

Between June 2021 and June 2024, 1417 fecal samples were collected from migratory birds in 12 sentinel points surrounding Nansi Lake in Jining City, China (Figure 1A and Supplemental Table S1), with an overall AIV positive rate of 2.1% (30/1417). And the prevalence of AIV was comparable for samples collected in 2023 (3.1%, 14/446) and 2024 (3.4%, 7/203), which was higher than those collected in 2021 (1.4%, 9/647), while no samples were positive for AIV in 2022 (Figure 1B and Supplemental Table S2). Of these 30 AIV-positive samples, four (13.3%), two (6.7%), and one (3.3%) were subtyped for H5, H9, and mixed virus, respectively, while no H7 subtype virus was identified (Supplemental Table S2). Subsequent sequencing and isolation identified one H5N6 and two H9N2 viruses from the fecal samples of migratory birds (Supplemental Table S4).

Meanwhile, a total of 978 samples were collected from 12 LPMs in Jining City, China, between July 2022 and June 2024 (Figure 1A and Supplemental Table S1). The overall prevalence of AIV among poultry samples was 10.2% (100/978) and the distribution of AIV varied by different quarters (Figure 1C and Supplemental Table S3). Further subtype analysis showed that the H9 subtype viruses were predominant, accounting for 35% (35/100) of the positive samples, followed by the H5 subtyped virus (30.0%, 30/100) and the mixed viruses (14.0%, 14/100), while H7 subtypes accounted for only 5% (5/100) of positive samples (Supplemental Table S3). Virus characterization identified two H5N1, seven H5N2, one H7N9, and three H9N2 subtypes, along with one N1 and two N2 subtype viruses lacking HA gene sequences (Supplemental Table S4). Notably, positive samples from migratory birds and poultry that failed subtyping using the standard H5/H7/H9 Detection Kit may represent non-H5/H7/H9 subtype infections, suggesting potential circulation of other AIV subtypes in this region (Supplemental Tables S2 and S3).

### 3.2. Phylogenetic and Reassortant Analysis of AIVs Among Migratory Birds and Poultry

**Figure 2:**
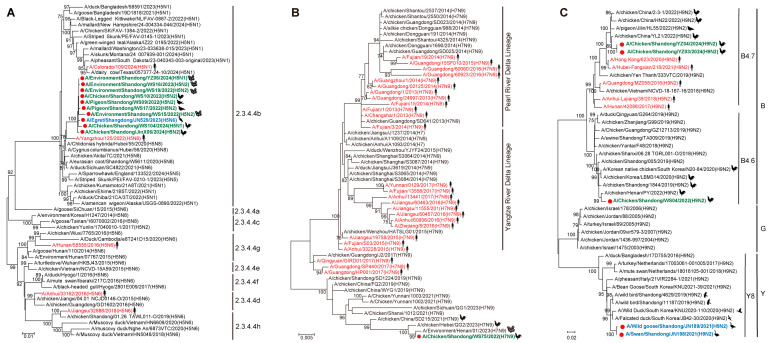
Phylogenetic analysis of hemagglutinin (HA) genes from influenza A viruses identified in this study. Maximum-likelihood phylogenetic trees were constructed for HA of H5 (**A**), H7 (**B**), and H9 (**C**) subtypes. Red dots indicate the viruses reported in this study. Green, blue, and red fronts indicate the viruses identified in poultry, migratory birds, and humans, respectively.

Phylogenetic analysis of the HA gene showed that all 10 sequenced H5 subtype viruses belonged to the HPAI H5 clade 2.3.3.4b, clustering closely with recent strains isolated from humans (e.g., A/Yangzhou/125/2022), wild birds, and poultry across China (Figure 2A). The HA gene of the H7N9 virus isolated in this study showed high similarity to contemporary poultry-origin H7N9 strains circulating in multiple Chinese provinces (Figure 2B). For H9N2 viruses, the HA of two viruses sequenced from poultry samples clustered with previous H9 clade B4.7 viruses identified in poultry and humans from Asia, and the other virus from poultry clustered with recent H9 clade B4.6 viruses clarified in poultry from Asia. However, two identified H9N2 viruses from migratory birds possessed the HA gene from the H9 clade Y8, which comprises exclusively wild bird isolates from Asia and Europe (Figure 2C).

**Figure 3:**
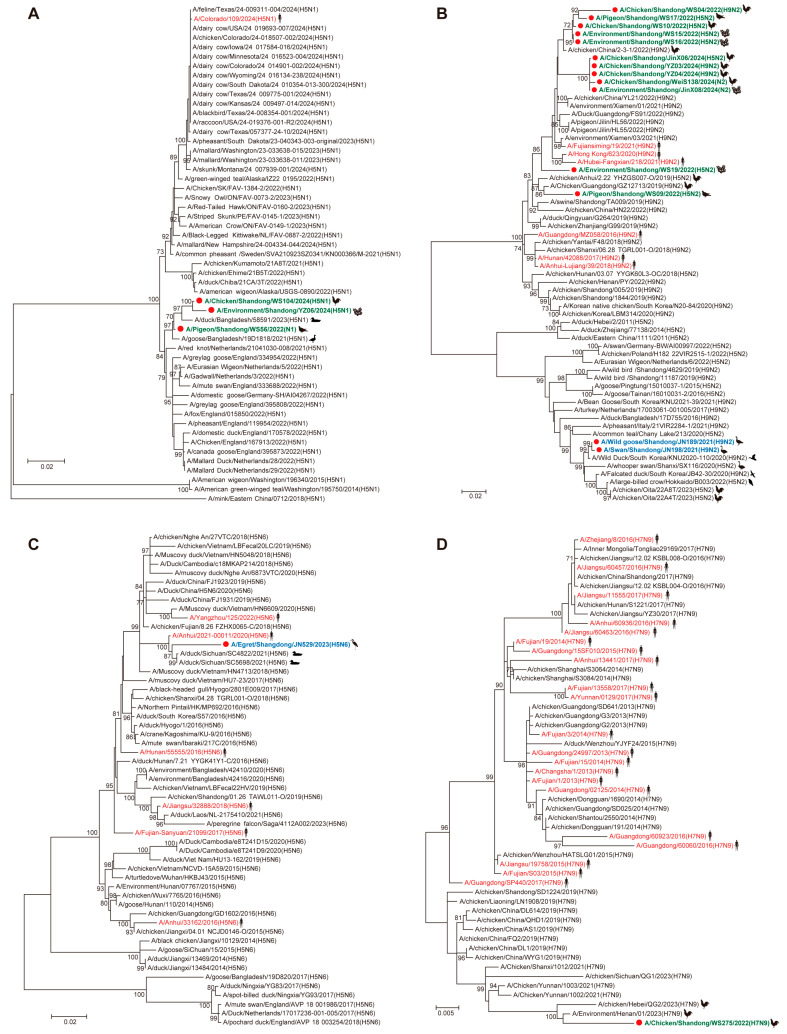
Phylogenetic analysis of neuraminidase (NA) genes from influenza A viruses identified in the study. Maximum-likelihood phylogenetic trees were constructed for N1 (**A**), N2 (**B**), N6 (**C**), and N9 (**D**) subtypes. Red dots indicate the viruses reported in this study. Green, blue, and red fronts indicate the viruses identified in poultry, migratory birds, and humans, respectively.

Phylogenetic analysis of NA genes revealed distinct clustering patterns among the identified viruses. All three N1 viruses clustered with contemporary H5N1 viruses identified in poultry from Bangladesh (Figure 3A). Notably, the NA genes of all poultry-origin H5N2 and H9N2 viruses in this study grouped within an established H9N2 lineage that included strains from both Chinese poultry and human cases (Figure 3B), while the two migratory bird-derived H9N2 viruses clustered with the recent H9N2 isolates identified in wild birds from multiple Asian countries (Figure 3B). The egret-origin NA gene of one H5N6 virus clustered with other H5N6 viruses identified in poultry, humans, and migratory birds across Asia (Figure 3C). The poultry-derived NA gene of one H7N9 virus clustered with other H7N9 viruses identified in Chinese poultry from several provinces (Figure 3D).

Phylogenetic analysis of the internal genes suggested that most of the H5 subtype viruses clustered closely with the human-origin H5N6 strain (A/Yangzhou/125/2022), while several viruses exhibited internal genes derived from the clade 2.3.4.4b H5N1-like virus (A/duck/Bangladesh/58591/2023) and the clade 2.3.4.4d H5N6-like virus (A/Anhui/33162/2016) (Supplemental Figure S1A–F). Similar to the surface genes, all the poultry-origin internal genes of the H7N9 virus clustered with the other Chinese H7N9 strains from several provinces (Supplemental Figure S2A–F). The poultry-origin internal genes of all H9N2 viruses grouped with other H9N2 viruses isolated in poultry and humans, while the two wild bird-derived H9N2 viruses possessed the PB2 gene from the H3N2-like virus (A/duck/China/402D27/2019), with the remaining internal genes probably originating from H9N2 lineages identified in Asian poultry and wild birds (Supplemental Figure S3A–F).

**Figure 4:**
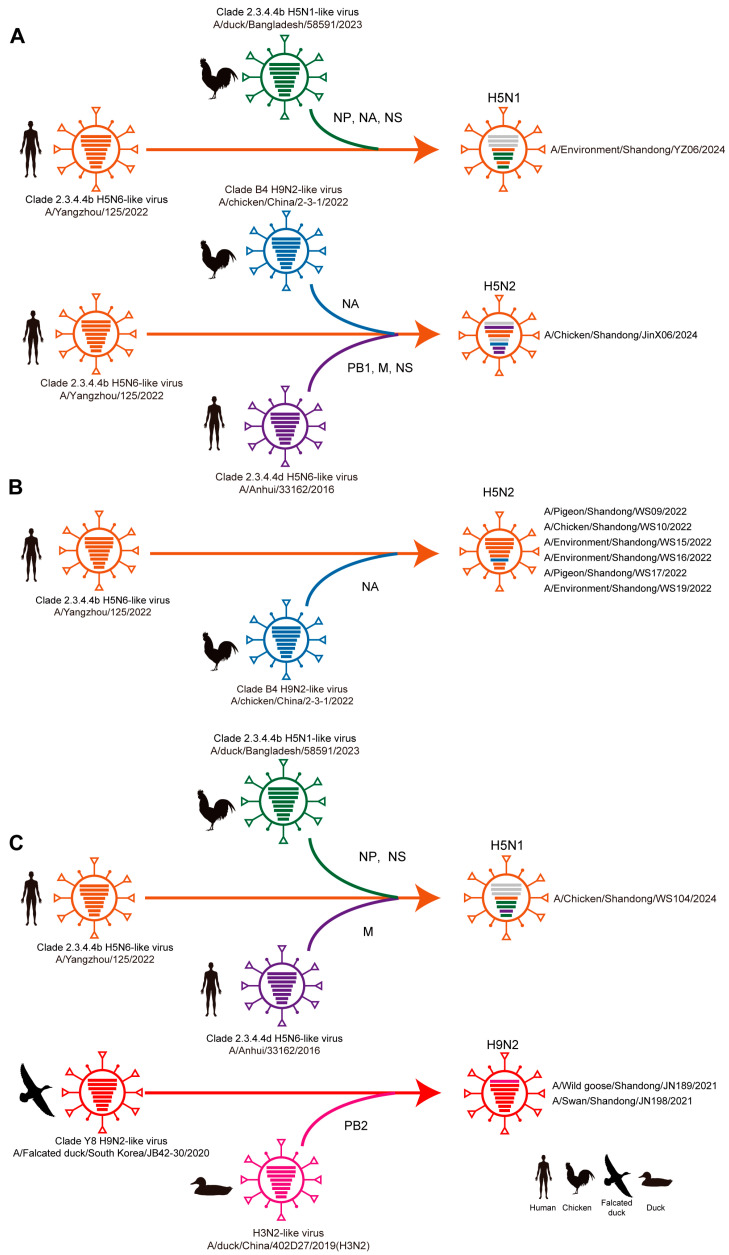
Probable genomic reassortment patterns of influenza A viruses identified in the study. Probable genomic reassortment patterns involving both surface and internal genes (**A**), only surface genes (**B**), and partial internal genes (**C**) of identified viruses in the study. Virus particles are represented by ovals containing horizontal bars that represent the eight gene segments (top to bottom: polymerase basic 2 (PB2), polymerase basic 1 (PB1), polymerase acidic (PA), hemagglutinin (HA), nucleoprotein (NP), neuraminidase (NA), matrix (M), and nonstructural (NS)). Colors indicate sequence origin based on initial viruses (gray bars indicate no sequence data available).

Further reassortment analysis revealed three distinct patterns among the studied AIVs (Figure 4A–C), using human-origin H5N6 (A/Yangzhou/125/2022) and wild bird-origin H9N2 (A/Falcated duck/South Korea/JB42-30/2020) as reference strains. First, whole-genome reassortment events involved both surface and internal genes (Figure 4A). For instance, the H5N1 strain (A/Environment/Shandong/YZ06/2024) acquired the NA, NP, and NS genes from a clade 2.3.4.4b H5N1-like virus (A/duck/Bangladesh/58591/2023), while the H5N2 strain (A/Chicken/Shandong/JinX06/2024) obtained the NA gene from a clade B4 H9N2-like virus (A/chicken/China/2-3-1/2022) and the PB1, NS, and M genes from a clade 2.3.4.4d H5N6-like virus (A/Anhui/33162/2016). Second, surface gene-limited reassortment was observed in six H5N2 strains (e.g., A/Pigeon/Shandong/WS09/2022), which exclusively acquired the NA gene from a clade B4 H9N2-like virus (A/chicken/China/2-3-1/2022) (Figure 4B). Third, partial internal gene reassortment occurred in the H5N1 strain (A/Chicken/Shandong/WS104/2024), which incorporated the NP and NS genes from a clade 2.3.4.4b H5N1-like virus (A/duck/Bangladesh/58591/2023) and the M gene from a clade 2.3.4.4d H5N6-like virus (A/Anhui/33162/2016) (Figure 4C). Additionally, two H9N2 strains (A/Wild goose/Shandong/JN189/2021 and A/Swan/Shandong/JN198/2021) acquired the PB2 gene from an H3N2-like virus (A/duck/China/402D27/2019) (Figure 4C).

### 3.3. Key Mutations of Identified AIVs Among Migratory Birds and Poultry

Molecular characterization revealed significant virulence markers across the studied avian influenza viruses (Supplemental Tables S5–S7). All H5 subtype viruses and one H7N9 virus had a polybasic amino acid residue at the cleavage site (EKRRKR/G or RKRAAR/G), confirming their highly pathogenic nature in avian species, whereas the H9N2 viruses showed low-pathogenicity cleavage motifs (PARSSR/G, PAASNR/G, or PSRSSR/G). All H5 subtype viruses had T160A mutations on the HA protein, indicating enhanced binding specificity for human-type receptors and increased transmissibility among guinea pigs [37]. All H9N2 viruses had T160A/N/S mutations, and three of them identified in poultry had E190V/A, T212I/V, and Q226L mutations on the HA protein, suggesting enhanced binding ability for human-type receptors and transmissibility among mammals [38]. All LPM-derived H9N2 viruses carried NA stalk deletions associated with enhanced virulence in mice, as well as increased adaptation and transmission in avian. One H7N9 virus had K526R, two H9N2 viruses had I292V, and one H9N2 virus had E627V mutations on the PB2 protein, revealing increased viral polymerase function and promoting viral replication in mammalian hosts. All H9N2 viruses had K356R and I550L mutations on the PA gene that are associated with increased mammalian replication and pathogenicity [39]. All H5 subtype viruses had N30D and T215A on the M1 protein, indicating increased virulence and polymerase activity in mammals [40]. V15I and A166V mutations were found on the M1 protein of H9N2 viruses, which were associated with host tropism and an increase in virulence [41]. Two H5 subtypes, one H7N9, and three H9N2 viruses had S31N mutations on the M2 protein, showing increased drug resistance against amantadine. All H9N2 viruses possessed the P42S mutation in the NS1 protein, enhancing viral virulence in mice [42]. F103I, I106M, and E227K mutations were also found on the NS1 protein of partial H9N2 viruses, which were associated with enhanced replication, virulence, and transmission [43].

### 3.4. Serological Investigation Among Avian-Exposed and General Population

We then conducted a serological investigation across four population groups, including the residents near migratory bird habitats (*n* = 687), the workers in poultry farms (PFs) (*n* = 104), the workers in LPMs (*n* = 144), and the general control population (*n* = 472), against each of the representative AIVs (Supplemental Table S8). We firstly screened the sera samples by the hemagglutination inhibition (HI) assay, with the HI titers ≥ 1:40 defined as positive (Table 1). The results showed that there were 6 (0.6%) subjects with positive HI results against the H5N1 strain, including 3 (0.04%) and 3 (2.1%) subjects in migratory birds-exposed group and LPM-exposed group, respectively. For the H5N6 strain, 1 (0.1%) and 4 (2.8%) subjects in the migratory bird-exposed group and LPM-exposed group had positive HI results, respectively. For H9N2 subtype, 13 (1.9%), 8 (7.7%), 4 (2.8%), and 1 (0.2%) subjects had positive HI results in the groups of migratory birds-exposed, PF-exposed, LPM-exposed, and the general control population, respectively. Furthermore, significantly higher positive prevalence of HI antibodies among the participants in the migratory bird-exposed group [Odds Ratio (OR) 9.1, 95% CI 1.5–97.2, *p* = 0.022], PF-exposed group (OR 39.3, 95% CI 5.9–436.6, *p* < 0.0001), and LPM-exposed group (OR 13.3, 95% CI 2.2–162.6, *p* = 0.014) was observed than that in the general control population. No positive HI results existed among the general control population against H5N1, H5N2, H5N6, and H7N9 subtypes. Further microneutralization (MN) assay confirmed that the PF-exposed subjects (OR 14.0, 95% CI 1.4–135.9, *p* = 0.003) and LPM-exposed subjects (OR 24.1, 95% CI 2.9–197.3, *p* < 0.0001) had an increased risk of getting an infection of the H9N2 subtype than the general population (Table 2). In addition, four (2.8%) and two (1.4%) subjects in the LPM-exposed group had seropositive results against the H5N1 and H5N6 viruses, respectively. Overall, the serological investigation demonstrates that H9N2, H5N1, and H5N6 subtypes may be more likely to re-emerge in humans than others among the selected AIV subtypes used for detection.

## 4. Discussion

In this study, we prospectively enrolled migratory birds, poultry, and exposed humans together to assess the risk of AIV transmission and human infections around Nansi Lake, Jining City, China. Sixteen AIVs, divided into five different subtypes (H5N1, H5N2, H5N6, H7N9, and H9N2), were identified in migratory birds and poultry. Genomic reassortment was elucidated among different AIV subtypes, as well as between migratory birds and poultry. Serological investigation suggested that both migratory birds and poultry-exposed populations had relatively higher seroprevalence against H9N2 than the general populations. Furthermore, we detected sporadic but concerning serological evidence of H5 and H7 subtype exposure among individuals with avian contact, revealing potential transmission risk of these AIV subtypes.

Migratory birds, as long-distance transmission media, spread the AIVs from one region to another, accelerating the process of transmission [44,45]. This was particularly evidenced by the two H9N2 viruses identified in wild birds, which showed high genetic homology with the viruses from different countries (South Korea, China, and Bangladesh), confirming the role of bird migration in facilitating the genomic reassortment of AIVs. Documented studies have found that migratory birds and poultry can share the gene segments pool of AIVs [46,47,48]. In this study, one H5N6 virus (A/Egret/Shandong/JN529/2023) was isolated from migratory birds and shared high similarities of HA and internal genes with six H5 subtype viruses identified in the surrounding LPMs, providing direct evidence of genomic reassortment of AIVs at the wild bird–poultry interface in the region of Nansi Lake. Notably, all gene segments of A/Egret/Shandong/JN529/2023 grouped closely with the human-origin H5N6 virus (A/Yangzhou/125/2022), suggesting these circulating H5 subtype viruses possess zoonotic potential.

LPMs are considered hubs for AIV transmission in poultry and are major risk factors for human infections [49,50]. Previous studies have found that the LPMs may increase the diversity of AIV subtypes through genomic reassortment among poultry with different origins, thus facilitating the emergence of public-health-threatening AIVs [16,51]. Consistent with these studies, four AIV subtypes, including H5N1, H5N2, H7N9, and H9N2, were co-circulating in the LPMs around Nansi Lake in our study. Furthermore, the HA gene of all H5 subtype viruses clusters in the same H5 clade 2.3.4.4b, in which those viruses have been widely detected in poultry, migratory birds, dairy cattle, other mammals, and even humans, posing a great threat to the breeding industry and public health [34,52,53]. Additionally, three different patterns of genomic reassortment were observed for H5N1 and H5N2 viruses, reflecting the two-way transmission of AIVs among poultry in LPMs. In September 2017, the application of an H5/H7 bivalent inactivated vaccine for chickens successfully controlled the prevalence of H7N9 among poultry and eliminated human infection with the H7N9 virus [54]. However, we found that antigenically altered H7N9 viruses were recently detected in many Chinese provinces, indicating that updated vaccines are needed to control the circulating H5/H7 viruses. The previous study has illustrated that the H9N2 viruses play an important role in generating novel avian influenza viruses through providing gene segments [3,55]. In this study, all H5N2 viruses probably possessed the NA gene from H9N2 viruses in the LPMs, suggesting that the control of H9N2 viruses in LPMs is also a key to controlling the prevalence of other AIV subtypes.

Indeed, documented studies have shown that the poultry-exposed population has a higher risk of getting infections of AIVs than the general control population [25,28,51]. Similarly, our study also shows that poultry-exposed subjects in both poultry farms and LPMs have higher seroprevalence against H9N2 than that of unexposed controls. Of particular concern, sporadic cases of infection with H5N1 and H5N6 are observed in LPMs, revealing the potential transmission risk of AIVs from poultry to humans. Notably, the residents near migratory bird habitats also exhibited higher seroprevalence against H9N2 than the general control population, underscoring the need for enhanced AIV surveillance in these ecologically vulnerable communities.

Overall, our study demonstrates that the genomic reassortment of AIVs frequently occurs between migratory birds and poultry, as well as among different AIV subtypes. Meanwhile, avian-exposed subjects, including both poultry workers and residents near migratory bird habitats, are at higher risk of AIVs infection than the general control population. These findings emphasize the urgent need for integrated One Health surveillance systems encompassing poultry, migratory birds, and exposed human populations to effectively monitor and mitigate emerging avian influenza threats.

## Figures and Tables

**Table 1:** Serological investigation of human infection with H5, H7, or H9 avian influenza A virus by hemagglutination inhibition (HI) assay.

Group	Subjects Tested No.	HI Titer ≥ 1:40 No. (%)					Odds Ratio (95% CI) ^a^	*p*-Value ^a^
		Anti-H5N1	Anti-H5N2	Anti-H5N6	Anti-H7N9	Anti-H9N2		
Avian-exposed subjects	935	6 (0.6)	0	5 (0.5)	0	25 (2.7)	12.9 (2.2–134.1)	0.003
Residents living around the habitat of migratory birds	687	3 (0.04)	0	1 (0.1)	0	13 (1.9)	9.1 (1.5–97.2)	0.022
Workers in poultry farms	104	0	0	0	0	8 (7.7)	39.3 (5.9–436.6)	<0.0001
Workers in live poultry markets	144	3 (2.1)	0	4 (2.8)	0	4 (2.8)	13.3 (2.2–162.6)	0.014
General population	472	0	0	0	0	1 (0.2)	Reference	Reference
Total	1407	6 (0.4)	0	5 (0.4)	0	24 (1.7)	/	/

CI, confidence interval. ^a^ Comparisons were performed between each of the avian-exposed groups and the general population group against H9N2.

**Table 2:** Serological investigation of human infection with H5, H7, or H9 avian influenza A virus by microneutralization (MN) assay.

Group	Subjects Tested No.	MN Titer ≥ 1:40 No. (%)					Odds Ratio (95% CI) ^a^	*p*-Value ^a^
		Anti-H5N1	Anti-H5N2	Anti-H5N6	Anti-H7N9	Anti-H9N2		
Avian-exposed subjects	935	4 (0.4)	0	2 (0.2)	0	20 (2.1)	10.3 (1.4–76.9)	0.005
Residents living around the habitat of migratory birds	687	0	0	0	0	10 (1.5)	7.0 (0.9–54.5)	0.032
Workers in poultry farms	104	0	0	0	0	3 (2.9)	14.0 (1.4–135.9)	0.003
Workers in live poultry markets	144	4 (2.8)	0	2 (1.4)	0	7 (4.9)	24.1 (2.9–197.3)	<0.0001
General population	472	0	0	0	0	1 (0.2)	Reference	Reference
Total	1407	4 (0.3)	0	2 (0.1)	0	21 (1.5)	/	/

CI, confidence interval. ^a^ Comparisons were performed between each of the avian-exposed groups and the general population group against H9N2.

## Data Availability

Data will be made available on request.

## Supplementary Materials

The following supporting information can be downloaded at https://www.mdpi.com/article/10.3390/v17081117/s1, Table S1. Information on samples collected from poultry, migratory birds, and humans in this study between 2021 and 2024 in Jining, China; Table S2. Avian influenza A virus surveillance in migratory birds between 2021 and 2024 in Nansi Lake of Jining, China; Table S3. Avian influenza A virus surveillance in live poultry markets between 2022 and 2024 in Jining, China; Table S4. Information on identified influenza A viruses in migratory birds and poultry; Table S5. Characterization of selected molecular markers associated with infectivity, pathogenicity, and antiviral susceptibility among H5 subtype viruses identified in the present study; Table S6. Characterization of selected molecular markers associated with infectivity, pathogenicity, and antiviral susceptibility against the H7N9 virus identified in the present study; Table S7. Characterization of selected molecular markers associated with infectivity, pathogenicity, and antiviral susceptibility against H9N2 viruses identified in the present study; Table S8. Demographic information of enrolled participants for serological investigation. Figure S1. Phylogenetic analysis of H5Nx internal genes; Figure S2. Phylogenetic analysis of H7N9 internal genes; Figure S3. Phylogenetic analysis of H9N2 internal genes.
