# Supplementary material for: Avian Influenza Surveillance Among Migratory Birds, Poultry, and Humans Around Nansi Lake, China, 2021–2024
Source: Viruses. 2025 Aug 14;17(8):1117. doi: 10.3390/v17081117 (PMC12390717; doi:10.3390/v17081117)
Supplement: Supplementary file 1 [file viruses-17-01117-s001.zip › viruses-3766457-supplementary.pdf]

Article

Avian Influenza Surveillance Among Migratory Birds, Poultry, and Humans Around Nansi Lake, China, 2021–2024

Table S1. Information of samples collecting from poultry, migratory birds, and humans in this study between 2021 and 2024 in Jining, China.

Sampling type	City	County	Sampling No.	Longitude and latitude
**Migratory bird habitat**				
MBH-1	Jining	Taibaihu	577	(116.6034E, 35.3196N)
MBH-2	Jining	Weishan	133	(117.1666E, 34.7403N)
MBH-3	Jining	Weishan	18	(116.6259E, 35.2684N)
MBH-4	Jining	Weishan	4	(116.8659E, 34.9345N)
MBH-5	Jining	Zoucheng	12	(116.9670E, 35.4155N)
MBH-6	Jining	Taibaihu	6	(116.4627E, 34.5841N)
MBH-7	Jining	Yutai	34	(116.8659E, 34.9345N)
MBH-8	Jining	Taibaihu	53	(116.5927E, 35.3128N)
MBH-9	Jining	Rencheng	10	(116.5134E, 35.2252N)
MBH-10	Jining	Yutai	35	(116.5621E, 34.9919N)
MBH-11	Jining	Weishan	220	(116.8659E, 34.9345N)
MBH-12	Jining	Weishan	315	(117.0879E, 34.7541N)
**Live poultry market**				
LPM-1	Jining	Wenshang	180	(116.4704E, 35.7145N)
LPM-2	Jining	Wenshang	10	(116.4926E, 35.7202N)
LPM-3	Jining	Jinxiang	144	(116.3101E, 35.0723N)
LPM-4	Jining	Yanzhou	20	(116.8237E, 35.5533N)
LPM-5	Jining	Yanzhou	90	(116.8251E, 35.6339N)
LPM-6	Jining	Zoucheng	124	(116.9910E, 35.4090N)
LPM-7	Jining	Liangshan	110	(116.0755E, 35.8031N)
LPM-8	Jining	Weishan	70	(117.1630E, 34.8283N)
LPM-9	Jining	Weishan	70	(117.0384E, 34.8878N)
LPM-10	Jining	Weishan	50	(117.1224E, 34.8683N)
LPM-11	Jining	Yutai	100	(116.6392E, 34.9916N)
LPM-12	Jining	Jiaxiang	10	(116.3304E, 35.4032N)
**Residents living around the migratory bird habitat**				
Humans-MBH-1	Jining	Weishan	95	(117.0764E, 34.8563N)
Humans-MBH-2	Jining	Weishan	84	(117.0913E, 34.8987N)
Humans-MBH-3	Jining	Weishan	87	(116.7431E, 35.1546N)
Humans-MBH-4	Jining	Weishan	97	(116.7247E, 35.1915N)
Humans-MBH-5	Jining	Weishan	123	(117.3770E, 34.6220N)
Humans-MBH-6	Jining	Weishan	201	(116.7247E, 35.1915N)
**Subjects working in the poultry farm**				
Humans-PF-1	Jining	Jinxiang	30	(116.0954E, 35.0364N)
Humans-PF-2	Jining	Zoucheng	25	(117.1980E, 35.3880N)
Humans-PF-3	Jining	Wenshang	25	(116.3516E, 35.6670N)
Humans-PF-4	Jining	Yanzhou	25	(116.6669E, 35.5577N)
**Subjects working in the live poultry market**				
Humans-LPM-1	Jining	Jiaxiang	20	(116.3304E, 35.4035N)
Humans-LPM-2	Jining	Liangshan	20	(116.0755E, 35.8031N)

Humans-LPM-3	Jining	Weishan	20	(117.1501E, 34.8329N)
Humans-LPM-4	Jining	Yanzhou	10	(116.8251E, 35.6339N)
Humans-LPM-5	Jining	Yanzhou	10	(116.8238E, 35.5533N)
Humans-LPM-6	Jining	Zoucheng	20	(116.9910E, 35.4090N)
Humans-LPM-7	Jining	Jinxiang	24	(116.3202E, 35.0436N)
Humans-LPM-8	Jining	Wenshang	10	(116.4705E, 35.7145N)
Humans-LPM-9	Jining	Wenshang	5	(116.4926E, 35.7202N)
Humans-LPM-10	Jining	Wenshang	5	(116.4956E, 35.7351N)
**General population**				
Humans-Control-1	Dezhou	Decheng	154	(116.3400E, 37.3504N)
Humans-Control-2	Dezhou	Decheng	157	(116.3190E, 37.4782N)
Humans-Control-3	Dezhou	Decheng	161	(116.3013E, 37.4658N)

Table S2. Avian influenza A virus surveillance in migratory birds between 2021 and 2024 in Nansi Lake of Jining, China.

Year	Sampling No.	Positive No. (%)	Subtype No. (%)				
			H5	H7	H9	Mixed	Un-subtyped
2021	647	9 (1.4)	0	0	2 (2.2)	1 (11.1)	6 (66.7)
2022	121	0	0	0	0	0	0
2023	446	14 (3.1)	1 (7.1)	0	0	0	13 (92.9)
2024	203	7 (3.4)	3 (42.9)	0	0	0	4 (57.1)
Total	1,417	30 (2.1)	4 (13.3)	0	2 (6.7)	1 (3.3)	23 (76.7)

Table S3. Avian influenza A virus surveillance in live poultry markets between 2022 and 2024 in Jining, China.

Year Quarter	Sampling No.	Positive No. (%)	Subtype No. (%)				
			H5	H7	H9	Mixed	Un-subtyped
2022							
3rd	60	12 (20.0)	7 (58.3)	0	1 (8.3)	0	4 (33.3)
4th	40	11 (27.5)	2 (18.1)	0	5 (45.5)	4 (36.4)	0
2023							
1st	46	15 (32.6)	0	2 (13.3)	12 (80.0)	1 (6.7)	0
2nd	100	7 (7.0)	0	0	0	0	7 (100)
3rd	160	16 (10.0)	12 (75.0)	0	0	3 (18.8)	1 (6.3)
4th	132	5 (3.8)	0	3 (60.0)	0	1 (20.0)	1 (20.0)
2024							
1st	360	18 (5.0)	5 (27.8)	0	5 (27.8)	5 (27.8)	3 (16.7)
2nd	80	16 (20.0)	4 (25.0)	0	12 (75.0)	0	0
Total	978	100 (10.2)	30 (30.0)	5 (5.0)	35 (35.0)	14 (14.0)	16 (16.0)

Table S4. Information of identified influenza A viruses in migratory birds and poultry.

Virus ID	Virus name	Sub-type	Isola-tion	Sequenced segments	Collection time	Host	Location
EPI_ISL_19450597	A/Wild_goose/Shandong/JN189/2021	H9N2	N	PB2, PB1, PA, HA, NP, NA, M, NS	2021-11-19	Wild goose	Weishan
EPI_ISL_19450593	A/Swan/Shandong/JN198/2021	H9N2	N	PB2, PB1, PA, HA, NP, NA, M, NS	2021-11-19	Swan	Weishan
EPI_ISL_19432679	A/Chicken/Shandong/WS04/2022*	H9N2	Y	PB2, PB1, PA, HA, NP, NA, M, NS	2022-08-09	Chicken	Wenshang
EPI_ISL_19429467	A/Pigeon/Shandong/WS09/2022	H5N2	N	PB2, PB1, PA, HA, NP, NA, M, NS	2022-08-09	Pigeon	Wenshang
EPI_ISL_19432670	A/Chicken/Shandong/WS10/2022	H5N2	N	PB2, PB1, PA, HA, NP, NA, M, NS	2022-08-09	Chicken	Wenshang
EPI_ISL_19433135	A/Environment/Shandong/WS15/2022	H5N2	N	PB2, PB1, PA, HA, NP, NA, M, NS	2022-08-09	Environment	Wenshang
EPI_ISL_19433136	A/Environment/Shandong/WS16/2022	H5N2	N	PB2, PB1, PA, HA, NP, NA, M, NS	2022-08-09	Environment	Wenshang
EPI_ISL_19432673	A/Pigeon/Shandong/WS17/2022*	H5N2	Y	PB2, PB1, PA, HA, NP, NA, M, NS	2022-08-09	Pigeon	Wenshang
EPI_ISL_19433137	A/Environment/Shandong/WS19/2022	H5N2	N	PB2, PB1, PA, HA, NP, NA, M, NS	2022-08-09	Environment	Wenshang
EPI_ISL_19433141	A/Pigeon/Shandong/WS56/2022	N1	N	NA, M, NS	2022-10-08	Pigeon	Wenshang
EPI_ISL_19450590	A/Chicken/Shandong/WS75/2022*	H7N9	Y	PB2, PB1, PA, HA, NP, NA, M, NS	2022-12-30	Chicken	Wenshang
EPI_ISL_19433139	A/Egret/Shandong/JN529/2023*	H5N6	Y	PB2, PB1, PA, HA, NP, NA, M, NS	2023-07-14	Egret	Taibaihu
EPI_ISL_19432676	A/Chicken/Shandong/JinX06/2024	H5N2	N	PB1, PA, HA, NA, M, NS	2024-06-20	Chicken	Jinxiang
EPI_ISL_19433143	A/Environment/Shandong/JinX08/2024	N2	N	PB1, NA, M, NS	2024-06-20	Chicken	Jinxiang
EPI_ISL_19432677	A/Chicken/Shandong/WS104/2024	H5N1	N	HA, NP, NA, M, NS	2024-06-18	Chicken	Wenshang
EPI_ISL_19450595	A/Chicken/Shandong/YZ03/2024	H9N2	N	PB2, PB1, PA, HA, NP, NA, M, NS	2024-06-25	Chicken	Yanzhou

EPI_ISL_19450596	A/Chicken/Shandong/YZ04/2024	H9N2	Y	PB2, PB1, PA, HA, NP, NA, M, NS	2024-06-25	Chicken	Yanzhou
EPI_ISL_19450594	A/Environment/Shandong/YZ06/2024	H5N1	N	HA, NP, NA, M, NS	2024-06-25	Environment	Yanzhou
EPI_ISL_19450599	A/Chicken/Shandong/WeiS138/2024	N2	N	NA, M	2024-06-26	Chicken	Weishan

*These isolates of avian influenza were selected for further serological investigation.

Table S5. Characterization of selected molecular markers associated with infectivity, pathogenicity, and antiviral susceptibility among H5 subtype viruses identified in the present study.

Virus name	Cleavage	HA*				NA		PB2			PA	M1		M2
		N158D	T160A	Q226L	G228S	R292K	Deletion	K526R	E627K	D701N	A343T	N30D	T215A	S31N
A/Pigeon/Shandong/WS09/2022(H5N2)	EKRRKR/G	N	A	Q	G	R	63-65	K	E	D	A	D	A	S
A/Chicken/Shandong/WS10/2022(H5N2)	EKRRKR/G	N	A	Q	G	R	63-65	K	E	D	A	D	A	S
A/Environment/WS15/2022(H5N2)	EKRRKR/G	N	A	Q	G	R	63-65	K	E	D	A	D	A	S
A/Environment/WS16/2022(H5N2)	EKRRKR/G	N	A	Q	G	R	63-65	K	E	D	A	D	A	S
A/Pigeon/Shandong/WS17/2022(H5N2)	EKRRKR/G	N	A	Q	G	R	63-65	K	E	D	A	D	A	S
A/Environment/WS19/2022(H5N2)	EKRRKR/G	N	A	Q	G	R	63-65	K	E	D	A	D	A	S
A/Environment/Shandong/YZ06/2024(H5N1)	EKRRKR/G	N	A	Q	G	R	63-65	K	E	D	A	D	A	S
A/Chicken/Shandong/JinX06/2024(H5N2)	EKRRKR/G	N	A	Q	G	R	63-65	K	E	D	A	D	A	N
A/Chicken/Shandong/WS104/2024(H5N1)	EKRRKR/G	N	A	Q	G	R	63-65	K	E	D	A	D	A	N
A/Egret/Shangdong/JN529/2023(H5N6)	EKRRKR/G	N	A	Q	G	R	63-65	K	E	D	A	D	A	S

*HA positions follow the H3 numbering. NSD, no sequence data.

Table S6. Characterization of selected molecular markers associated with infectivity, pathogenicity, and antiviral susceptibility against the H7N9 virus identified in the present study.

Virus name	Cleavage	HA*					NA		PB2			PA		NP	M2	NS1
		N158D	T160A	E190D	Q226L	G228S	R292K	Deletion	K526R	E627K	D701N	S37A	D383N	E210D	S31N	I106M
A/Chicken/Shandong/WS275/2022	RKRAAR/G	N	T	E	Q	G	R	69-73	R	E	D	S	D	E	N	I

*HA positions follow the H3 numbering.

Table S7. Characterization of selected molecular markers associated with infectivity, pathogenicity, and antiviral susceptibility against H9N2 viruses identified in the present study.

Virus strains	Cleavage	HA					NA		PB2			PA		M1		M2	NS1			
		T160A	E190V	T212I	Q226L	G228S	R292K	Deletion	I292V	E627K/V	D701N	K356R	I550L	V15I	A166V	S31N	P42S	F103L	I106M	E227K
A/Chicken/Shandong/WS04/2022	PARSSR/G	A	A	V	L	G	R	63-65	I	V	D	R	L	I	A	N	S	L	I	K
A/Chicken/Shandong/YZ03/2024	PAASNR/G	N	V	I	L	G	R	63-65	V	E	D	R	L	I	A	N	S	L	I	K
A/Chicken/Shandong/YZ04/2024	PAASNR/G	N	V	I	L	G	R	63-65	V	E	D	R	L	I	A	N	S	L	I	K
A/Wild goose/Shandong/JN189/2021	PSRSSR/G	S	E	T	Q	G	R	NO	I	E	D	K	L	V	V	S	S	F	M	E
A/Swan/Shandong/JN198/2021	PSRSSR/G	S	E	T	Q	G	R	NO	I	E	D	K	L	V	V	S	S	F	M	E

*HA positions follow the H9 numbering.

Table S8. Demographic information of enrolled participants for serological investigation.

Characteristics	Residents living around the habitat of migratory birds	Workers in poultry farms	Workers in live poultry markets	General population
Total No.	687	104	144	472
Age, median (IQR)	59.0 (47.0-70.0)	47.0 (38.0-55.0)	52.5 (44.3-58.0)	50.0 (36.0-62.0)
Gender				
Male	301 (43.8)	53 (51.0)	73 (50.7)	231 (48.9)
Female	386 (56.2)	51 (49.0)	71 (49.3)	241 (51.1)

IQR, interquartile range.

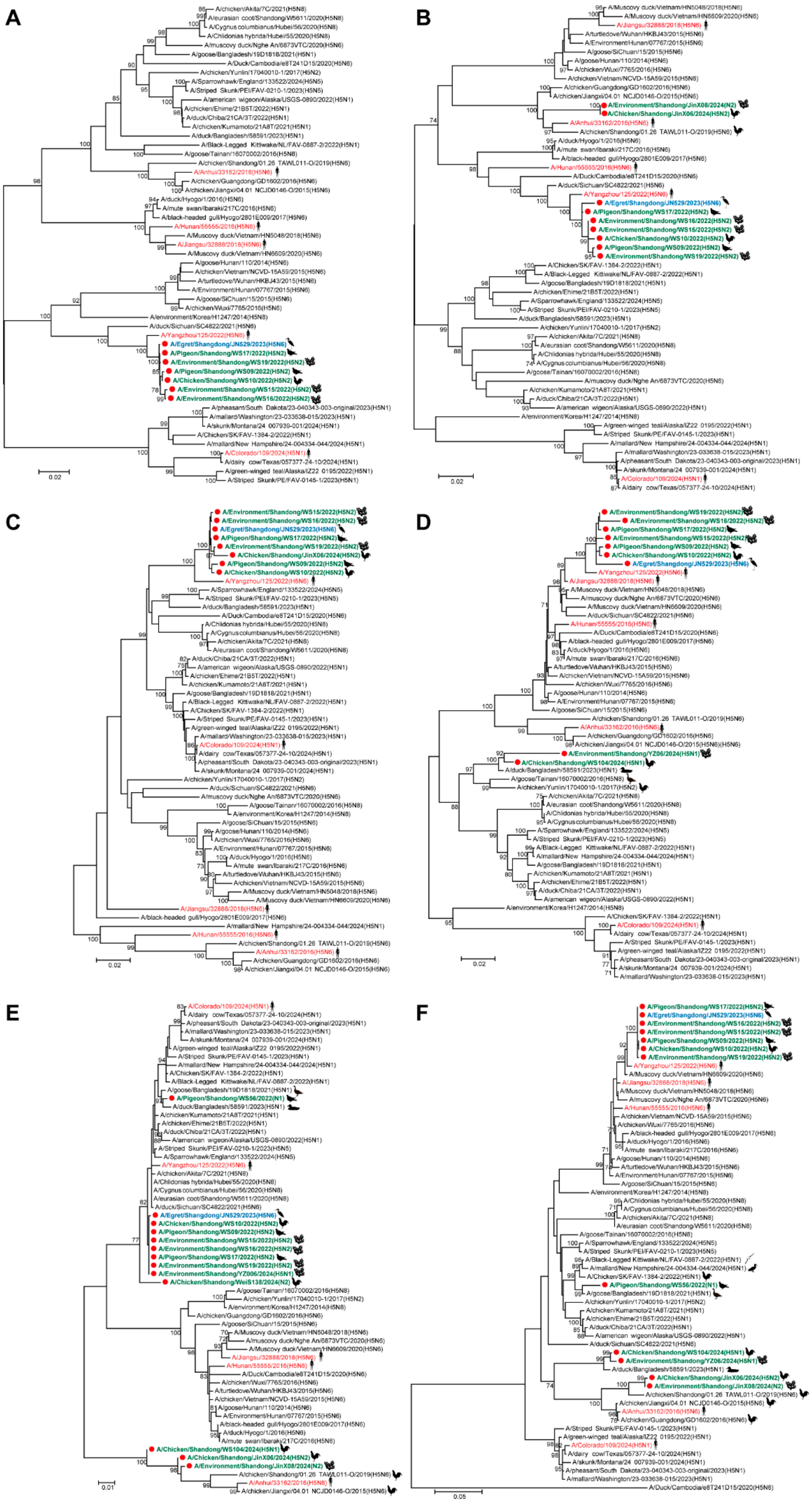

Figure S1. Phylogenetic analysis of H5Nx internal genes. Maximum-likelihood phylogenetic tree for PB2 (A), PB1 (B), PA (C), NP (D), M (E), and NS (F) of H5N1, H5N2, and H5N6 viruses identified in this study. Red dots indicate the viruses reported in this study. Green, blue, and red fronts indicate the viruses identified in poultry, migratory birds, and humans, respectively.

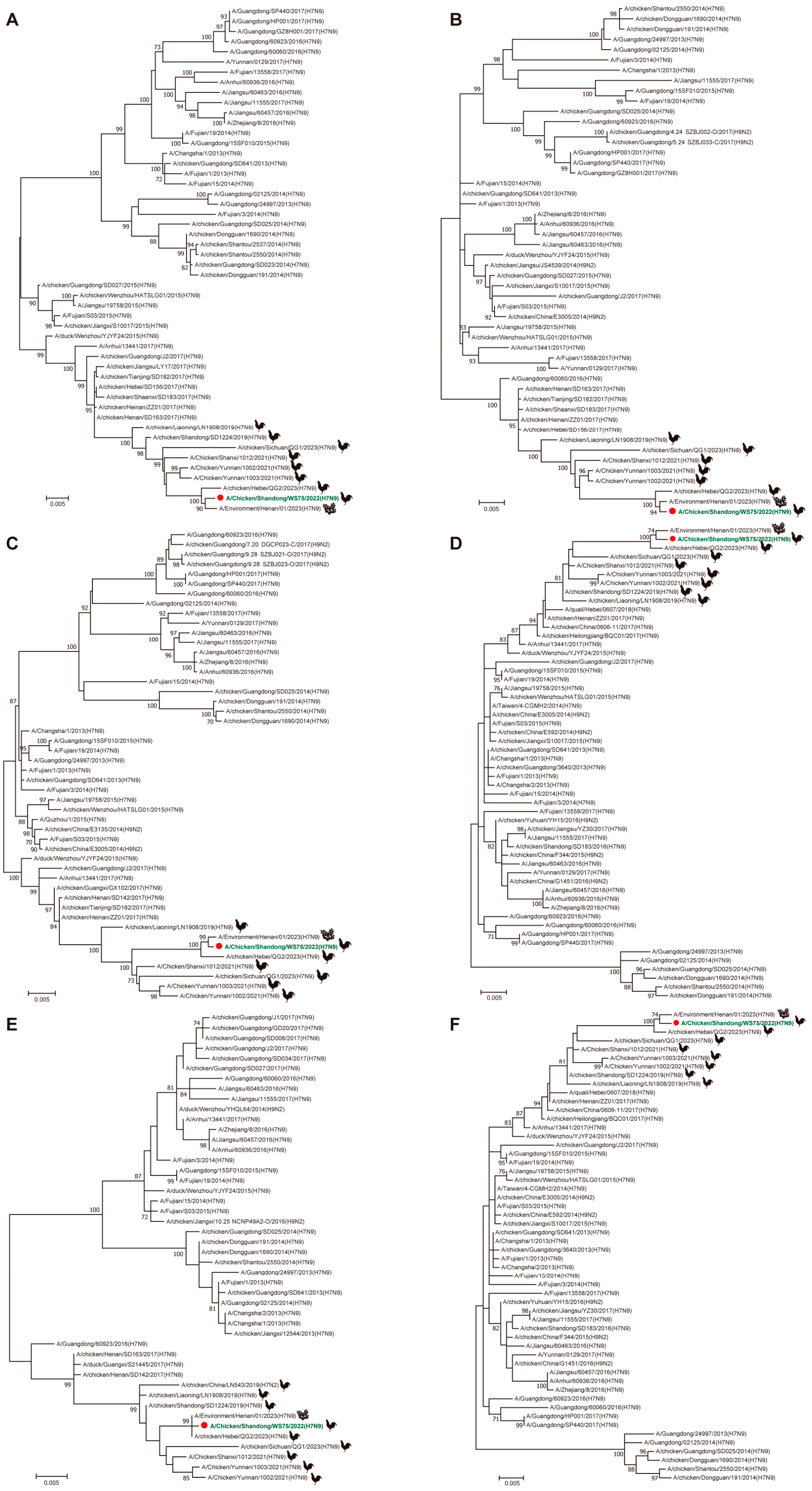

Figure S2. Phylogenetic analysis of H7N9 internal genes. Maximum-likelihood phylogenetic tree for PB2 (A), PB1 (B), PA (C), NP (D), M (E), and NS (F) of H7N9 viruses identified in this study. Red dots and green front indicate the virus identified in poultry in this study.

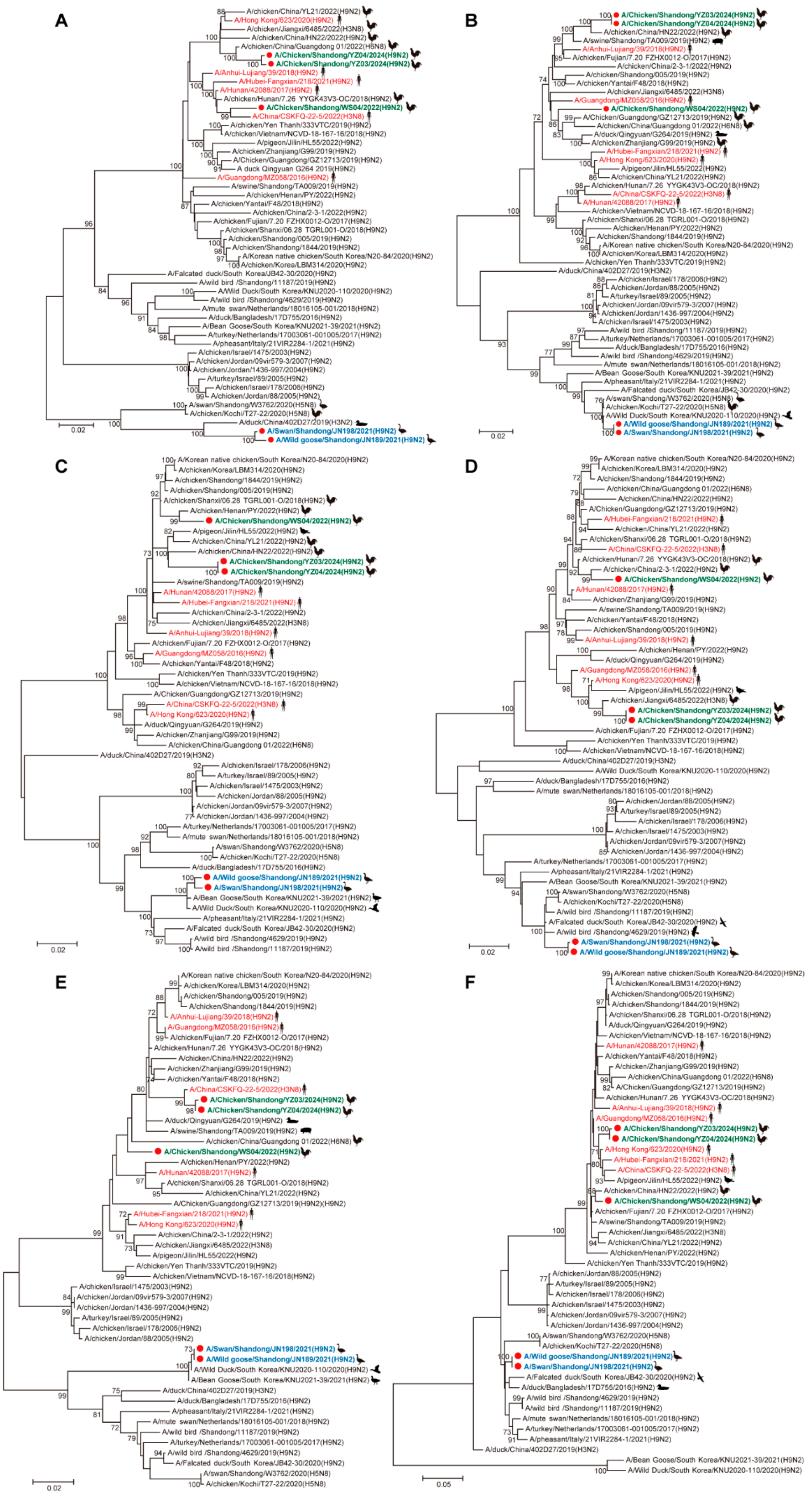

Figure S3. Phylogenetic analysis of H9N2 internal genes. Maximum-likelihood phylogenetic tree for PB2 (A), PB1 (B), PA (C), NP (D), M (E), and NS (F) of H9N2 viruses identified in this study. Red dots indicate the viruses reported in this study. Green, blue, and red fronts indicate the viruses identified in poultry, migratory birds, and humans, respectively.
